# Ophthalmology

**Published:** 1896-12

**Authors:** Alvin A. Hubbell

**Affiliations:** Professor of ophthalmology at Niagara University Medical College


### OPHTHALMOLOGY.

CONDUCTED BY ALVIN A. HUBBELL, M. D.,
Professor of ophthalmology at Niagara University Medical College.

### THE FORMATION OF ARTIFICIAL PUPIL BY EXTRAOCULAR IRIDOTOMY.

AT A MEETING of the British Medical Association recently, (*British Medical Journal*, September 26, 1896,) an address by J. B. Lawford, F. R. C. S., of St. Thomas's Hospital, London, was given on The formation of artificial pupil by extraocular iridotomy, in which he states :

That the operative procedure, variously described as extraocular iridotomy or precorneal iridotomy, was introduced by Professor Schoeler, of Berlin, and described by him in 1886.

Mr. Lawford has performed the operation on a limited number of patients since 1891, and has, so far, found it a satisfactory method of dealing with cases in which, for various reasons, it is desirable to make a small coloboma in the iris, for visual purposes.

The cases for which it seems most suitable are corneal opacity, involving the central area, and leaving the periphery, in whole or in part, transparent ; stationary nuclear opacity of the lens, and obstruction of the pupil by deposit on the anterior lens capsule, as a result of disease or as a congenital defect.

It is, he thinks, preferable to intraocular iridotomy (except in aphakic eyes), because safer, and superior to iridectomy in its optical results.

The position in which the gap in the iris should be made will necessarily vary. Generally, the inferior nasal quadrant has been selected ; want of transparency of the cornea in this position may, however, interfere, and the coloboma should then be made in the lower or lower and outer part. The technique of the operation, in his hands, is as follows : A small corneal incision is made with a triangular keratome, close to, but not quite at, the limbus cornea; the knife is carefully withdrawn to avoid a following prolapse of iris. A pair of fine, curved iris forceps, without teeth, is then introduced, and the iris gently grasped close to its pupillary border and withdrawn. It is divided at right angles to the pupillary edge and through about half its width with a pair of iris scissors, and afterwards carefully and gently returned to the anterior chamber.

After tucking back the iris, eserine drops may be applied. The eye is then tied up with a pad and bandage, and the patient kept perfectly quiet for twenty-four hours.

The whole proceeding should be carried out with the strictest precautions against sepsis. As a rule, no reaction follows and the corneal wound heals rapidly. He has had no serious iritis in any case. In adults, generally, cocaine produces sufficient anesthesia; in children a general anesthesia is necessary.

---

### LOCATION OF FOREIGN BODIES IN THE EYE WITH RÖNTGEN RAYS.

Dr. Clark, of Columbus, O., at the thirty-second annual meeting of the American Ophthalmological Society, held at New London, Conn., July, 1896, reported a case in which the presence of a small fragment of metal in the extreme angle of the anterior chamber and the iris, where it could not be seen, had been determined by skiagraphy. The sensitive plate had been introduced into the adjoining nostril, the patient being put under ether, and the rays directed upon it through the eye from the temporal side. He suggested that the plate could also be placed in the cocainised conjunctival sac, or an opening could be made in the conjunctiva and the small plate slipped behind it. He believed that this method of locating a foreign body in the eye-ball was perfectly practicable, especially if the particle were lodged anteriorly, as in the ciliary region, where it could not be seen with the ophthalmoscope.

Dr. C. H. Williams, Boston, Mass., reported a case in which a fragment of the copper case of a cartridge had passed through the cornea and lens. Nothing could be seen of it and it was not certain that it was in the eye. The use of the X-rays showed the presence of the fragment and it was removed. The skiagraph was obtained with ten minutes' exposure by laying the patient's head with the side of the injured eye upon the plate, and placing the Crookes' tube above and rather in front of the patient's head.

---

### CATARACT EXTRACTION.

G. A. Berry, (*British Medical Journal*, Sept. 26, 1896,) in discussing the subject of cataract extraction at a recent meeting of the British Medical Association, said :

I used to think the only advantage of simple overcombined extraction was the round pupil, a cosmetic advantage to which, on account of the age of the cataract patients as a rule, and of the fact that the cornea is usually to some extent covered by the lid, I was not prepared to ascribe much importance, especially if a small coloboma be made when the combined operation is done. I now consider—and, indeed, I am inclined to go further and say I know—that there are other and more important advantages in a simple extraction if the operation be performed by the method to which I shall presently refer.

On the other hand, there are unquestionably greater difficulties in the simple operation itself, and certain cases in which it is more or less unsuitable. A proper course, therefore, that is, one calculated to give the best results in a large number of cases, necessitates making a selection. If it were necessary to choose between the two operations, one which should be put in practice in every case, I should unhesitatingly choose the operation with iridectomy. That operation, too, I should recommend to all whose experience of extracting for cataract is limited. Hence, in talking of making a selection, I mean selecting the cases in which one may attempt to get the greater advantages of the simple operation with the best chances of success.

The method of performing simple extraction which has led me to alter my views as to its value is one which, so far as I know, is due to Prof. Snellen. At all events, I have only seen it put in practice by him. The incision is large, occupying half the circumference of the cornea. It lies in the apparent corneo-scleral margin, and is made with a large and broad conjunctival flap, the knife being carried for fully a quarter of an inch below the conjunctiva before cutting out after the section at the corneo-scleral margin is completed. The cystotome is introduced from the side, that is, at the one end of the incision. The cornea is then pressed upon below the center, so as to cause the large wound to gape considerably and make the edge of the lens escape in front of the iris. A strong solution of pilocarpine is used immediately before operating. Needling is done a fortnight later.

Snellen operates without a speculum, the lids being held apart by a good assistant. He also fixes the conjunctiva at a point opposite the point of puncture. The site of the incision at the corneo-scleral margin secures good opposition, and the large conjunctival flap provides well for the vitality of the cornea and for rapidity of healing. The incision, though large, has not, therefore, any of the objections which led to the abandonment of the corneal flap of the earlier simple extractions. To come now to the question of iris prolapse, the bugbear, if one may judge from many different reports of those who make it a rule to practise simple extraction by other methods. I have no hesitation in saying that prolapses are so rare when the operation is done in the manner I am now discussing that they need not practically be considered to occur at all. This would seem to some a pretty bold assertion, and, indeed, it does require to be qualified in one important way. Prolapses do occur, but they occur only during, and sometimes within an hour or two after operation, but not later. Since discovering this, I have made it a rule to perform iridectomy whenever the iris does not at once go back of itself or by rubbing on the cornea, or after running the shell repositor along the wound, and go back so completely as to leave a perfect round and contracted pupil.

No doubt iridectomy at this stage will be necessary more often at the hands of one operator than of another. At present I have to resort to it in about one case out of ten, and I believe it is more prudent to overdo this precaution than otherwise. It is well, too, to see the eye in other cases a short time after operation, especially where the patient has been allowed to walk from the operation table to bed. It is comparatively rare to find a prolapse then, if the strong solution of pilocarpine has been used, but if found it should be removed and not replaced.

With these exceptions, then, I say prolapse does not occur. Even the risk of a slight blow to the eye, which is not so uncommon an occurrence and which often leads to the bursting of the wound and obliteration of the chamber in the ordinary operation with iridectomy, need not be feared. The wound, owing to rapidity of healing of the conjunctival flap, is so much stronger that should it give way at all it does so over a comparatively small extent.

### THE THERAPEUTIC VALUE OF HYDROBROMATE OF SCOPOLAMINE IN PLASTIC IRITIS.

C. A. OLIVER (*American Journal of Medical Sciences*, November, 1896,) sums up his experience with hydrobromate of scopolamine as follows :

1. Hydrobromate of scopolamine is of the greatest value in the local treatment of the various forms of plastic iritis.

2. The primary reparative action and quieting power of hydrobromate of scopolamine, as compared with those of similar doses of sulphate of atropine in the treatment of plastic iritis, are much more promptly obtained with the former than with the latter drug, this being true in the majority of cases even when the latter drug is used in dosages that are equal to quadruple or quintuple the strengths that are employed of the former drug.

3. The duration of the healing and soothing powers of hydrobromate of scopolamine, as compared with those of equal doses of sulphate of atropine in cases of plastic iritis, does not seem to be so lasting with the former as with the latter drug, this being true in the majority of cases even when the latter drug is used in four or five times the strength doses as the former.

4. For quick and active measures, which are so eminently necessary in incipient cases of plastic iritis and during the early stages of inflammatory reaction, hydrobromate of scopolamine is to be preferred to sulphate of atropine.

5. Where prolonged use of such drugs is necessary, as in many cases of the chronic form of the disease with subacute exacerbations, the alternate employment of hydrobromate of scopolamine and sulphate of atropine seems empirically to be the best method of local administration that has been devised.

6. As critically employed, the best salt of the alkaloid seems to be the hydrobromate ; the best method of instillation appears to be that which is accomplished by dropping the solution upon the corneal limbus whilst the lower punction is everted and the corresponding canaliculus is pressed upon, and the most efficient amount to be used at one sitting is two drops of a one-tenth of 1 per cent. strength (1 to 500), repeated, if necessary, as often as three times during the course of an hour, and preceded, when desired, as in some instances where there are much irritation and pain, by two drops of a 2 per cent. solution of hydrobromate of cocaine a few minutes before each instillation of the scopolamine.

### UPON THE VALUE OF EUCAINE IN OPHTHALMIC PRACTICE.

RICHARD VOLLERT (*Annals of Ophthalmology and Otology*, July, 1896,) has used eucaine, a drug which has been recently brought forward by the chemical factory of Actien (formerly Schering) as a new local anesthetic. Eucaine has, like cocaine, a very long chemical name and formula (Amethyl-benzoyl-tetramethyl-gamma-oxypiperedine-carbonic acid methylester) $C_{19}$ $H_{27}$ $No_4$ CHL $H_2$ O. The, muriate is in shining plates and also crystallises in shining prisms from mythalic alcohol. Both are soluble to 6 per cent. in water at ordinary temperature. The base is with difficulty soluble in water, but with HCL makes a very soluble salt which remains anesthetic when heated, and is also soluble in 1-5 per cent. sublimate solution. When dropped in the eye in a 5 per cent. solution it produces painful irritation, lasting for one or two minutes (longer than cocain and more irritating). Considerable injection of the conjunctival and ciliary vessels, with first lessened and later heightened tension, follows. Anesthesia of the cornea and conjunctiva is secured in two to three minutes, lasting for eight to twelve minutes, and disappears entirely in fifteen minutes, causing little or no action upon the pupil or accommodation. Eucaine, like cocaine, impairs the corneal epithelium, perhaps more than the latter. On account of its feeble action in producing mydriasis and paresis of the accommodation, it may be useful as an anesthetic in selected cases. Vollert, however, does not think that our old friend, cocaine, will be superseded by this new drug, principally on account of the irritative effect of the latter.

Vinci, also, has experimented with this new compound from the laboratory of Prof. Liebreich. He finds that instilled into the eye of an animal in 2 to 5 per cent. solution causes complete local anesthesia in from one to three minutes, beginning with the cornea, spreading to the conjunctiva, and lasting on an average from ten to twenty minutes ; pupil does not dilate. Injected under the skin or painted on the .mucosa, complete anesthesia follows. General action as a poison shows excitation on the central nervous system, followed by paralysis, going on to death.

Eucaine is, he says, physiologically similar to cocaine, but is less poisonous. It causes no ischemia ; on the contrary, vascular dilation occurs, and in ophthalmic practice it may be employed where the former is not desired. In the eye the pupils are not affected ; mydriasis does not occur and reaction to light remains normal. The drug was tried in Prof. Schweigger's ophthalmological department at the University clinic, in Berlin, with the same result. Patients complain of transitory burning, but only when the methyl-alcohol preparation was used ; therefore the watery solution is to be preferred for practical use. Cocaine and eucaine diminish the intraocular pressure, but equally. Eucaine solutions are permanent and do not, like those of cocaine, decompose when kept or when boiled.

---

### PHOTOGRAPHY OF THE RETINA.

TH. GUILLOZ (*Medical Week*, June 5, 1896,) says that he first successfully employed photography of the retina for clinical purposes in the year 1893. His procedure is based on the following principle: When the pupil is dilated, the fundus of the eye may be illuminated, so as to permit of examination of the retina with a lens, without the necessity of any ophthalmoscopic mirror. The observation is thus made on the reversed image, and it is this image which is fixed by means of a photographic objective. Moreover, as the time of exposure, however short, is an inconvenience, he has constructed a special apparatus for instantaneous photography of the retina.

The photographs obtained in this manner show the ophthalmological image reversed, as it appears on examination, with the reflection from the optic disc and cornea ; but since then the author, by a new method of illumination, has succeeded in getting rid of this reflection.

---

### A NEW METHOD OF TREATING PTERYGIUM.

ANTON COE, of Washington, D. C., (*Annals of Ophthalmology and Otology*, April, 1896,) says he is satisfied that in the treatment of pterygium, the whole trouble lies alone in the apex, and that the vascular supply is not a part of the disease, but only an effect, as it but answers the call of the apex for blood, and, thus reasoned, that if the apex could be destroyed the vascular supply would cease from disuse.

The eye being placed under the influence of a 2 per cent. solution of cocaine, applied to the apex only, he heats to redness a platinum probe and applies it to the lower half of the apex, leaving the upper and deeper portions for the third and fifth days. In one case reported the patient was seen again on the third day, when it was found that the vessels feeding the lower portion first cauterised were disappearing, and they so progressed until six days after, when there was scarcely a trace of the former trouble.

---

### PROGNOSTIC VALUE OF ALBUMINURIC RETINITIS.

Possaner, (*Beitr. z. Augenheilk.*, *Heft* XV., 1895 ; *Annals of Ophthalmology and Otology*, April, 1896,) after studying the records of 67,000 patients seen in Haab's clinic in Zurich, finds that albuminuric retinitis was observed in only 131 cases (1.9 per cent.). Authors vary greatly as regards frequency of retinitis in kidney disease : 6.7 per cent. (Schweigger) to 33 per cent. (Galezowski). Most writers agree that, as a rule, death occurs within two years at least after appearance of the retinitis. Possaner has seen seventy-two cases, thirty-nine in private and thirty-three in hospital practice. Of the latter there were fourteen men, all of whom died within two years ; of the nineteen women, thirteen died within two years, two after a longer time, and four were still alive. The longest period of life observed was six years. Of the thirty-nine private patients (twenty-six men and thirteen women), sixteen men and seven women died within two years. In those still living the retinitis is dated from a minimum of two and a quarter to a maximum of eleven years. The better surroundings of private patients give them advantages, but even here the mortality is 53 per cent. in the first two years.

---

### ON THE PHYSIOLOGICAL ACTION OF ARECOLIN, A NEW MYOTIC ALKALOID.

Lavagna, (*Annals of Ophthalmology and Otology*, January, 1896,) while investigating the physiological effects of the salts of arecolin, found them to possess very energetic, though temporary, myotic qualities. One drop of a 1 per cent. solution produced, in five minutes a well-marked contraction of the pupil, which lasted seventy minutes. This was accompanied by a spasm of the ciliary muscle, similar to that set up by eserine. It also lowered the tension of glaucomatous eyes treated by it. Lavagna recommends its employment in all cases where a thorough, but temporary, myosis is desired.

